# Primary care visits increase utilization of evidence-based preventative health measures

**DOI:** 10.1186/s12875-020-01216-8

**Published:** 2020-07-28

**Authors:** Jeffrey Hostetter, Nolan Schwarz, Marilyn Klug, Joshua Wynne, Marc D. Basson

**Affiliations:** 1 Department of Family and Community Medicine, School of Medicine & Health Sciences, University of North Dakota, Grand Forks, USA; 2 School of Medicine & Health Sciences, University of North Dakota, Grand Forks, USA; 3 Department of Population Health, School of Medicine & Health Sciences, University of North Dakota, Grand Forks, USA; 4 Department of Internal Medicine, School of Medicine & Health Sciences, University of North Dakota, Grand Forks, USA; 5 Department of Surgery, School of Medicine & Health Sciences, University of North Dakota, Grand Forks, USA

**Keywords:** Primary care, Vaccination, Colonoscopy, Mammography, Wellness visits

## Abstract

**Background:**

Primary care visits can serve many purposes and potentially influence health behaviors. Although previous studies suggest that increasing primary care provider numbers may be beneficial, the mechanism responsible for the association is unclear, and have not linked primary care access to specific preventative interventions. We investigated the association between the number of times patients accessed their primary care provider team and the likelihood they received selected preventative health interventions.

**Methods:**

Patients with complete data sets from Sanford Health were categorized based on the number of primary care visits they received in a specified time period and the preventative health interventions they received. Patient characteristics were used in a propensity analysis to control for variables. Relative risks and 95% confidence intervals were calculated to estimate the likelihood of obtaining preventative measures based on number of primary care visits compared with patients who had no primary care visits during the specified time period.

**Results:**

The likelihood of a patient receiving three specified preventative interventions was increased by 127% for vaccination, 122% for colonoscopy, and 75% for mammography if the patient had ≥ 1 primary care visit per year. More primary care visits correlated with increasing frequency of vaccinations, but increased primary care visits beyond one did not correlate with increasing frequency of mammography or colonoscopy.

**Conclusions:**

One or more primary care visits per year is associated with increased likelihood of specific evidence-based preventative care interventions that improve longitudinal health outcomes and decrease healthcare costs. Increasing efforts to track and increase the number of primary care visits by clinics and health systems may improve patient compliance with select preventative measures.

## Background

Affordable and effective health care has proven difficult to achieve. Many efforts to make health care less expensive and more effective strive to shift care to primary care providers assuming primary care-based health care delivery will be more efficient and thus cheaper when administered by generalists treating a broad gamut of disease [1, 2]. This would seem prudent since primary care has evolved to be more focused on patient needs and the health of the population that it serves. It is thus more person-centered than disease-centered with primary care providers serving as the preferred access point to the health system, providing continuity and coordination of care over a person’s lifetime, and giving comprehensive care to the whole person [3, 4]. Thus robust primary care should keep people more healthy which obviously benefits patients, but should also benefit the health system in terms of decreasing cost and utilization. Indeed, many markers of population health are improved in areas with more primary care physicians [5]. Some, but not all, studies suggest that receiving health care from a primary care physician improves health outcomes [5–8]. Differences among populations studied could contribute to the observed inconsistency among population-based studies [5–8]. The relationship between primary care and improved outcomes seems strongest in rural areas, but is more inconsistent in urban populations and among Medicare beneficiaries [6, 8–10]. Such improvements in outcomes have resulted in predictions that over 100,000 deaths could be prevented annually by increasing access to primary care physicians [11]. The majority of literature on the benefits of primary care is based on studies done in the United States, and findings may differ when data from other countries and health systems are analyzed.

Most studies describing the benefits of primary care point to the lower overall cost of providing health care to a population and attribute these savings to care provided by family medicine physicians [5, 9]. But a crucial question--even if primary care is more cost-effective than specialist-based care [5]— is whether it improves health outcomes? We observed that the influence of primary care on rates of compliance with primary preventative care interventions has been less well investigated, and the limited available studies have found mixed results [9, 12–14]. Similarly, while many previous studies show correlations between increasing primary care providers and decreasing population morbidity and mortality, the available studies did not follow people over time to establish a link between primary care and outcomes [6, 10, 11, 15] While such data are available in the electronic health record databases of health systems, a clear link between the provision of care and outcomes has not been widely published [15].

We hypothesized that an increased number of primary care visits would be associated with increased frequency of patients receiving primary preventative care interventions that have been demonstrated to improve health outcomes. Since access is a key component of the primary care relationship, we sought to correlate receiving specific preventative interventions with primary care access patterns. Specifically, we investigated the correlation between the number of times each patient accessed his or her primary care team and the likelihood of receiving one of three pre-specified preventative care interventions. We studied colonoscopy, mammography, and vaccinations because they are well-established as having significant beneficial impacts on patient morbidity and mortality [16–21], and recommendations from advisory medical organizations for these three have remained relatively consistent. Evidence for benefit from an annual wellness visit (AWV) is equivocal [22, 23]; however, since they have been emphasized by some insurers and health care organizations, and are typically performed by primary care physicians, we thought that it would be interesting to study the rate of compliance with AWVs as well.

## Methods

### Sanford health

The Sanford Health system covers a large area of the upper mid-western United States, and includes urban, rural, and frontier clinics and hospitals. At the time of data collection, it served 2.74 million people with 44 medical centers and 291 clinics across four states. The system utilizes the Epic electronic health record system and has a central electronic repository that stores patient data from all of its hospitals, clinics, and care delivery sites.

### Data

Under a protocol approved by Sanford Health and University of North Dakota institutional review boards, we received an aggregate data file from the Sanford Data Collaborative abstracted from records for all patients using the Sanford system between January 2014 and December 2016. Patients who accessed Sanford only during the last six months of 2016 were excluded to permit sufficient follow up. Patients with unreported age, age over 100, or missing any values were also excluded. We chose an age limit of 100 because there is likely benefit to even nonagenarians receiving vaccinations and we had data in the very elderly within the dataset to analyze. Few mammographies or colonoscopies were done in patients over 75, consistent with current thinking that the harm-benefit ratio for screening mammography or colonoscopy may be unfavorable in many very elderly patients. The data used to count visits was billing data based on CPT and E&M codes. Thus, the numbers for vaccinations, mammograms and colonoscopies could be excluded from provider visits. Only provider visits were counted.

Although Sanford had 2.7 million patients in their data system, we only received active 1,143,028 records for the 3-year period of the study. Other patients registered in the system may have moved, died, or simply not sought any medical care during that 3-year period. Because we were not granted access to the entire 2.7 million patient data set, we cannot distinguish whether the active patients were in some way different from completely inactive patients. Within the data that we were able to analyze, some records were deleted because their age was not in the infant to 100 range (*n* = 881). 192,015 were missing BMI but after regression imputation only 319 were missing. Some other variables with missing data included gender (337), race (37,377), marital status (14,680), and urban/rural status (3054). These and other variables were such a small percentage of the total number of other records that bias was unlikely. Hospitalizations, clinic visits, and immunizations were considered none if the field was left blank. We therefore used 1,039,227 of the possible records, available to us, eliminating 103,801 (9%) for incomplete information. The database that we received for analysis was custom-created for our research by the Sanford Health System and had linked billing, procedural and compliance data to primary care visits so that we were able to determine for each patient recorded whether the patient had undergone primary care visits (and how many) and whether the patient had been coded as having colonoscopy, mammography, and/or vaccinations.

The 1,039,227 patients with complete data were separated into four subgroups that met pre-specified criteria for analysis. We analyzed vaccinations for all patients. Vaccinations were recorded as number per year and categorized as none versus one or more per year, and as having 0, 1, 2, 3–4 and 5 or more per year. We considered only patients age 50 and older in analyzing colonoscopy, only female patients age 50 and older for mammography, and only patients age 65 and older for AWVs. Colonoscopies, mammograms, and AWVs were measured as binary events occurring at least once at Sanford during the study period. Primary care visits per year were categorized as none, one, two, and three or more, and were defined as any visit, regardless of purpose, with a primary care doctor (family medicine, general internal medicine, or pediatrics), physician assistant, or nurse practitioner.

### Propensity analysis

Several patient characteristics (age, sex, race, rural status, smoking status, and alcohol use) were correlated with primary care visits. Health care utilization characteristics correlated with primary care visits included whether the patient used the hospital, the emergency room, or the MyCare system (an electronic patient portal). Appendix describes these characteristics. Propensity analysis predicting primary care visits determined an inverse weight that was then applied in the statistical analyses to control for the specified variables.

### Statistical analysis

Relative risks with 95% confidence intervals were used to estimate the likelihood of obtaining preventive measures by patients who had one or more primary care visits in a year versus those with none. Risks were further estimated for the subgroups of patients who had one, two, or three to four, or five or more visits per year all relative to patients with no visits. Confidence intervals rather than significance tests were used to describe the magnitude of the increase likelihood of obtaining preventive measures. The inverse weight determined by the propensity analysis was applied to all risk estimations to control for the patient characteristics.

## Results

### Vaccinations

We studied 1,039,227 patients over a three-year period for the number of vaccinations and primary care visits received per year. There was large variability in the number of vaccinations received by individual patients in a given year. In order to determine appropriates ranges for analysis, we used the CDC guidelines for children and adult vaccinations which could result in a child getting up to 24 vaccinations in a year and an adult getting 20 vaccinations. All data were controlled for rural versus urban demographics and remained statistically significant. The likelihood of having received one or more vaccinations per year was increased by 126% if a patient had one or more primary care visits per year (Table 1). The likelihood of having received one, two, three to four, and five or more vaccinations increased by two to six times.

**Table 1:** Relative risks of obtaining vaccinations if the patient had one or more primary care visits (relative to no primary care visits), sorted by age

Age Group	Number or Vaccinations per Year									
	1 or More		1		2		3 or 4		5 or More	
	RR	95% CI	RR	95% CI	RR	95% CI	RR	95% CI	RR	95% CI
**All Ages**	2.26	2.25–2.27	2.54	2.52–2.56	4.00	3.96–4.04	4.83	4.77–4.88	6.02	5.95–6.10
**Infant**	1.33	1.28–1.38	2.45	1.30–4.64	1.67	1.37–2.03	2.49	1.84–3.37	1.46	1.38–1.54
**1–10**	1.93	1.91–1.94	2.58	2.62–2.64	3.37	3.28–3.46	3.65	3.57–3.74	3.71	3.64–3.78
**11–19**	1.95	1.93–1.97	2.43	2.37–2.50	3.01	2.92–3.10	3.48	3.39–3.56	4.78	4.65–4.91
**20–60**	2.19	2.18–2.21	2.30	2.27–2.32	3.61	3.56–3.67	4.62	4.53–4.71	5.69	5.53–5.85
**61 or Older**	2.71	2.69–2.74	3.38	3.33–3.44	5.90	5.77–6.02	7.56	7.38–7.74	8.29	7.80–8.61

N = 1,039,227

The likelihood of a patient who had at least one primary care visit having received a vaccination increased with age (Table 1, column ‘1 or More’). The risk ratio (RR) of a newborn having received any number of vaccinations was 1.33, for children ages one to ten RR = 1.93 and for children ages eleven to 19 RR = 1.95. The risk was more than doubled for patients aged 20 to 60 and nearly tripled for those over 60. This increase in likelihood was similar when tested for patients having just one vaccination per year (RR = 2.45 to 3.38), two vaccinations (RR = 1.67 to 5.90), three to four (RR = 2.49 to 7.56), and five or more (RR = 1.46 to 8.29).

**Fig. 1:**
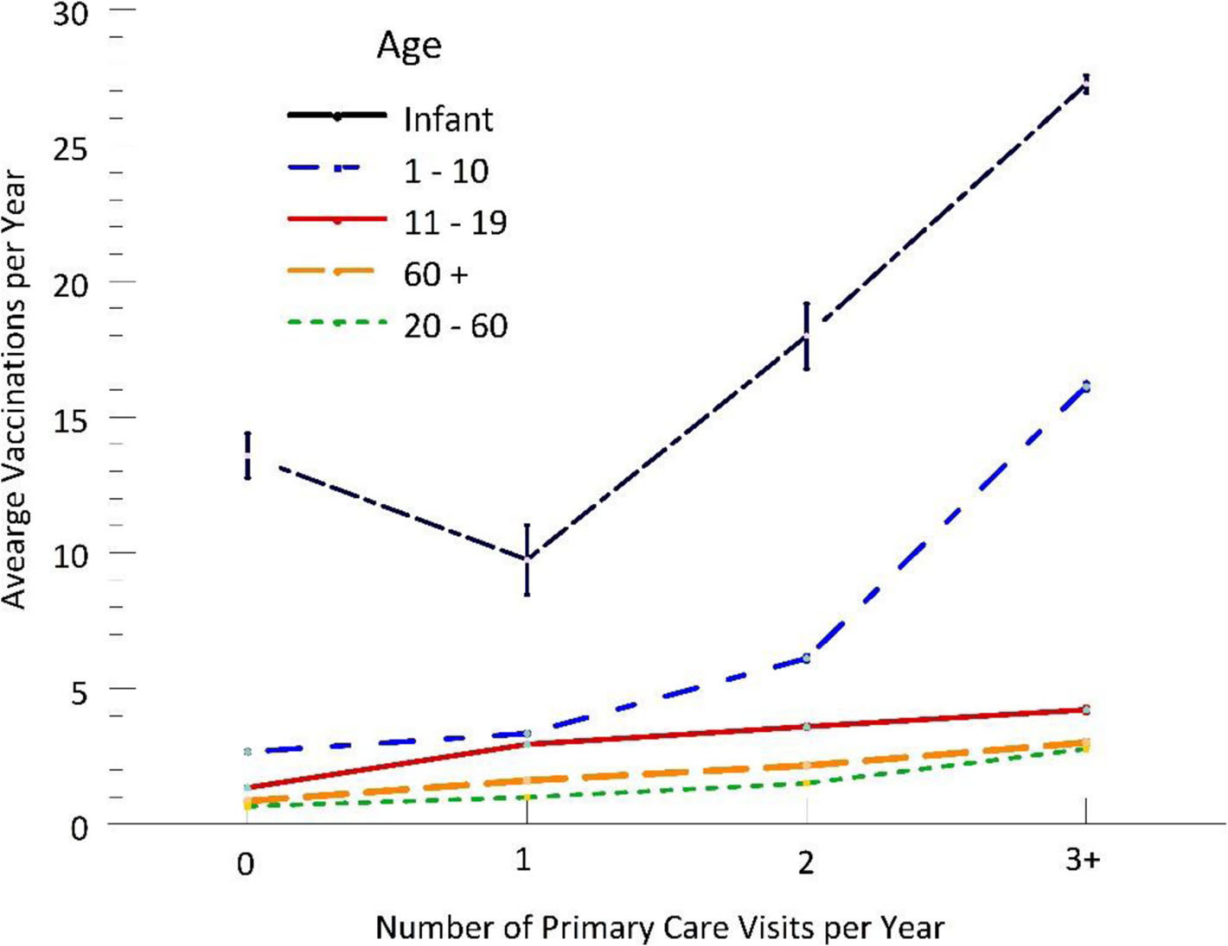
Average number of vaccinations per year by number of primary care visits per year

Patients with one or more primary care visits per year had twice as many vaccinations on average for all age groups (Fig. 1 bars, t = 129.1, t = 110.2, t = 101.9, all *p* < .001). Children had nearly three times as many vaccinations per year. Figure 1 also shows the fairly linear increase for the total number of vaccines in the 20 to 60 and 61+ age groups (F = 11,552, F = 4908, all p < .001). The increase was more dramatic in children, from 2.1 vaccines per year with zero visits to 15.2 per year with three or more (F = 42,748, p < .001).

### Colonoscopy, mammograms, annual wellness visits

The results of these analyses are shown in Table 2. The likelihood of having received a colonoscopy also correlated with having one or more primary care visits per year and increased the likelihood of having received a colonoscopy by 122% when compared to patients with no primary care visits. Although patients who had 3 visits or more were slightly more likely to have colonoscopy than patients who had only one visit, this was not statistically significant.

**Table 2:** Relative risks of colonoscopies, mammograms, and wellness visits if patient had a primary care visit relative to no primary care visits

Number of Primary Care Visits	Colonoscopy		Mammogram		Wellness Visit	
	RR	95% CI	RR	95% CI	RR	95% CI
1 or More	2.22	2.21–2.24	1.75	1.73–1.76	2.98	2.67–3.32
1	2.08	2.06–2.10	1.71	1.70–1.72	4.26	3.77–4.81
2	2.35	2.33–2.37	1.79	1.78–1.80	2.92	2.54–3.35
3 or More	2.36	2.34–2.38	1.76	1.75–1.77	1.67	1.43–1.96

Colonoscopy: N = 371,200 ages 50 and older

Mammogram: N = 200,147 females ages 50 and older

Wellness Visit: N = 174,655 ages 65 and older

For the eligible women studied, 67.9% had mammograms, and 49.1% of them had one or more primary care visits per year. Having one or more primary care visits per year increased the likelihood of having a mammogram by 75%. Patients who had more than one visit were not more likely to have mammography than patients who had only one visit.

Only 1% of the patients had received an AWV, and of that group 48.4% had received one or more primary care visits per year. During the study period, an AWV and a primary care visit could not be performed at the same visit or on the same day. Thus, a patient could have had an AWV as a preventative measure without having a primary care visit counted in the data. The likelihood was increased by nearly three-fold with primary care visits. This likelihood was four times greater for patients with only one primary care visit per year and, interestingly, decreased over 200% as primary care visits increased.

## Discussion

Although the cost benefits of primary care have been well-established in the literature, the impact of such care on quality has been less well examined. The literature, which includes confounding population-based studies, has demonstrated conflicting results as to the impact of primary care on general populations [5, 10, 11]. Our results suggest that increased engagement with primary care improves patient compliance with at least three evidence-based preventative interventions: vaccination, colonoscopy, and mammography. Our findings also raise the possibility that primary care visit usage in a population may be a surrogate marker for population health risk.

One global issue that we addressed was how to count visits and what visit fit into what category. Since the Sanford health system operates its own health insurance plan, the data set was constructed from visit data as well as billing data. Thus, it was possible to separate provider visits from procedure visits in order to avoid double counting. For instance, a mammogram would not be counted as a primary care visit since it is associated with a specific procedure code while the visit that was associated with the referral for the mammogram was associated with an evaluation and management (E&M) code. This is similarly true for vaccination and colonoscopy data. Additionally, since the compliance with preventive measures is based on billing data, this did not preclude the possibility that the visit associated with the measure was counted. This is true, because a referral could have come from a non-Sanford provider or the vaccination could have been given at a public health department or pharmacy. In all of these cases, however, a bill would have been sent for the procedure by the pharmacy, health department, or radiology department, and thus the intervention would be included in the data. In these cases, the resulting effect would have been to weaken the association between the primary care visit numbers and the risk of receiving the intervention. Finally, since the Sanford health system is a complete health network, it is unusual for patient to go outside the network for their care. For example, it would be unlikely for an infant delivered by a Sanford doctor to receive their vaccinations at a pharmacy or public health department.

Two other possible sources of discrepancies that we were unable to determine from the data would be vaccinations received at Indian Health System (IHS) or tribal clinics, and the possibility of self-referral for mammogram. Tribal and IHS clinics would not have submitted bills unless the patient was covered under a Sanford health plan. The number of American Indians who receive their health care at these clinics would be a very small portion of the data: 1–2% at most. While it is possible to self-refer for mammogram in one of the four states covered by the Sanford health system, this is rarely done due to liability issues. It was not possible for us to calculate exactly how much any of these issues attenuated the effect found in data. This may be a subject for future study.

Vaccination rates were significantly increased by one primary care visit and further affected by increasing visits. The benefit of vaccinations to reduce disease burden, hospitalization, mortality, and cost is well documented [20, 21, 24, 25]. Thus, our results suggest that increasing primary care visits will be associated with improved downstream vaccination-associated health outcomes. One of the few previous studies looking directly at connections between primary care visits and preventative measures was by Fiscella and Holt, who observed lower effects on vaccination than other preventative measures, while we found primary care’s effect on vaccinations to be the most robust of the specified interventions [26]. However, Fiscella studied only influenza vaccination rather than all vaccinations. Many patients appear to be more resistant to the yearly influenza vaccine than to other vaccinations, perhaps because of widespread publicity about the variable efficacy of the influenza vaccine in each year. This might explain the attenuated effect of primary care visits on vaccinations in Fiscella’s study. Our broad focus on all vaccinations more accurately represents the goal to translate primary care visits to health outcomes because much of the outcome improvement reflects other vaccines in addition to influenza [20, 25]. Fiscella [26] also studied a population only 1.5% of our study size with a narrower demographic(age 65+ Medicare beneficiaries).

The likelihood of receiving a colonoscopy also increased with more primary care visits, albeit more modestly than was the case with vaccinations. Since screening colonoscopy cost-effectively reduces colorectal cancer mortality [16, 19, 27], it is disappointing that primary care visits did not increase the rate of receiving colonoscopy as strongly as vaccination. Different from vaccination, we observed a large increase in colonoscopy screening with a single visit but relatively smaller increases with subsequent visits. While patients can be vaccinated at the time of their clinic visit, colonoscopy is harder to arrange and generally less attractive to patients. Patients willing to have colonoscopy may have been offered it at their first visit, so rates at subsequent visits might include a subgroup of patients who resist either the concept or the process. It is also possible that physicians who knew patients had declined colonoscopy at the first visit prioritized other concerns on subsequent visits. This trend aligns with Fiscella’s study [26], although Fiscella found colon cancer screening to be more affected than vaccination. Fiscella studied only patients over 64 [26]. We studied patients over 49 following American Cancer Society colorectal screening guidelines at that time. Younger patients may be less concerned with colon cancer, and less compliant than Medicare patients 65 and older. Although the inclusion of these younger patients may have reduced the impact of primary care visits on colonoscopy, their inclusion depicts the correlation between primary care visits and health more accurately across a broad population. In addition, the Medicare database used in Fiscella’s study also gave screening credit for fecal occult blood testing and barium contrast enema [28]. The barriers to these tests are distinctly different from the barriers to colonoscopy and could have contributed to the differences between the studies.

We also found the increase in likelihood of receiving a mammogram seemed to plateau at two primary care visits. Previous studies using Medicare data also found increased mammography associated with primary care visits, but did not demonstrate the plateau that we observed [18, 26, 28, 29]. Since previous studies were based on Medicare patients, the plateau we observed after the second visit might reflect higher initial compliance rates (RR of 1.71 for the first visit vs. 1.39 [26]), and/or older patients being more resistant overall, but more responsive to additional visits (RR of 1.76 for the third visit vs. 1.48 [26]).

In our study, the likelihood of the AWV [30] peaked at one primary care visit. Having more than one primary care visit led to a paradoxical decrease in AWV. This could be explained by the fact that most people are generally healthy, with a yearly hospitalization rate of only 7.6% [30], and require few visits of any kind. Whereas multiple primary care visits may have reflected chronic health concerns, which may have precluded interest in and time for AWV. An AWV was able to be distinguished from a primary care visit and not counted twice, because an AWV has a separate procedure code from an office visit code, and during the study period, they could not be performed at the same visit or on the same day.

The findings in our study raise the possibility that the number of primary care visits, a metric easily captured in electronic medical records, can be used to gauge the risk of individuals to not receiving recommended preventative care measures. Numerous complex studies have sought to identify patients who are at risk for specific high cost or high mortality conditions [31, 32]. The data collected for such studies typically include sicker patients on average [33] and results were analyzed independent of outcome [34]. Using primary care visits as a gauge likely would maintain clinical utility in patients who are more representative of the overall population. This measure also could be useful for primary care quality improvement efforts that frequently have focused only on increasing and quantifying the degree of access to primary care [5, 9, 11, 35]. But increasing potential access does not guarantee increasing use, and could be a major source of ambiguity in such research.

Our results from a large health system, covering a broad population, address limitations of previous smaller, more specific population-based studies [5, 8, 35]. We retrospectively analyzed a broad unselected population including both rural and urban areas, which facilitates extrapolation of these findings to large populations at the health system and clinic level. The findings remained statistically significant even after controlling for rural versus urban demographic data; however, it should be acknowledged that the population studied did not include areas of extremely high population density. Most inconclusive studies on the benefits of primary care examined dense urban populations while regional studies have shown positive benefits [5, 8–10]. Thus, it remains possible that primary care visits affect high-density populations differently; perhaps because urban patients are more likely to self-refer to specialists, bypass primary care providers, and thus not receive as many preventative health interventions.

Some have questioned whether regular primary care visits are needed at all. Healthcare resources must be allocated judicially whether at the microallocation or macroallocation levels [36]. A Cochrane review found evidence suggesting that regular health checks for healthy patients provide no benefit [37]. While our findings link primary care visits to preventative measures, others point out that these visits also build trusting and healing relationships [38]. The ambiguity about the benefit of primary care could reflect a complex effect between undistinguished types of primary care visits including direct illness management, developing trusting relationships, preventative screening, or combinations of the three. The effect of all three together could create a synergistic effect leading to the well-documented benefits of primary care [5, 7, 9, 13] whereas analyzing one aspect, such as in the Cochrane review, may miss some of the benefit and thus interpret primary care delivery as unnecessary.

During the study period, patients might have been insured in different ways that might have required that patients pay varying amounts out of pocket to defray some of the costs of these preventative measures, depending on their plan and how much they might have already paid over the course of the year in other health care costs. ACA-compliant plans covered preventative care with no costs, while “grandfathered plans” typically required patients to pay at least some part of the cost of colonoscopy or mammography while generally still covering vaccinations. The effect of patient co-pays on compliance with health care has been studied previously [39, 40], but is beyond the scope of the current study.

Our study had some limitations. The first is the potential for bias. The patients who we studied are the patients who go to the doctor or to the hospital, and thus have a propensity to seek medical care. We did not capture data from patients who had not engaged with the Sanford system at all over the three-year period of the study. Although many may have moved or died prior to the study period, some may simply not have sought any medical care. There is a possibility that this correlates with a certain personality type, type of insurance coverage, or other factor that is not captured in the data analyzed. There is no way to ascertain if the correlations observed were actually caused by primary visits or some of these other factors. Similarly, we eliminated 9% of our patient population for missing data. While there is no particular reason to hypothesize that those with incomplete data were different from those with complete data, we cannot establish equivalency without having their data, and this represents another source of potential bias. Furthermore, our data does not allow us to distinguish patients receiving primary care who came to the physician seeking that preventive care (e.g. vaccination) from patients who received primary care and were “prescribed” the preventive care during that visit since we cannot adjust for user lead vs physician lead prescription.

Second, we are unable to definitively link specific visits to specific interventions. Indeed, some patients may have received interventions due to referrals from providers outside the Sanford system, while conversely patients may have received interventions at hospitals outside the Sanford system that resulted from a primary care visit from a Sanford provider. The latter, seems unlikely, however, since we captured data from the Sanford insurance plan as well as system medical records, and mammography, colonoscopy, or vaccination outside the Sanford system would most probably still have been billed to the Sanford health insurance plan.

Another issue pertains to the data itself. There is no way to definitively link a specific visit to receiving a specific intervention. Indeed, some patients may have received interventions due to referrals from providers outside the Sanford system, and patients may have received interventions at hospitals outside the Sanford system that resulted from a primary care visit from a Sanford provider. In this last instance, however, the intervention would likely have been captured in the data unless the patient’s insurance was not billed for the procedure. Next, in the case of vaccinations, there is no way to know definitively if the patient came in solely for vaccination or came in for a primary care visit and received their vaccinations as part of the visit. However, we did not count nursing visits as a primary care visit, and most vaccination-only visits are billed as nursing visits. Other questions might also be raised that are not answerable by the data available. For example, we were unable to correct for morbidity which might be associated with both visits and immunizations, we were unable to distinguish between screening mammography or colonoscopy and diagnostic or therapeutic procedures, either in the outpatient or inpatient setting, and we could not control for additional potential confounders such as median income or insurance status or past patterns of care utilization. Future research might seek to use an instrumental variable approach to tease this out, but the necessary data for such approaches were not available to us. Finally, there is no way to quantify the effect of health education received at a primary care visit in the cases where patients received preventative care without referral from primary care. This effect would be most pronounced in the vaccination data, as the liability of performing mammogram or colonoscopy without referral is a barrier preventing this from being a widespread practice. Additionally, vaccinations do not have as many possible complications as mammogram or colonoscopy so a referral for these services requires more time for discussion of risks versus.

## Conclusions

In summary, one or more primary care visits per year are associated with an increased rate of patient compliance with evidence-based guidelines for preventative health interventions that have been shown to be directly related to improvements in health outcomes. The three-year span of this study was too short to measure effects such as reduced prevalence and/or mortality, and the data set lacked information to specific intermediate effects such as disease-specific risk reduction. This awaits future study. Increased effort to track and encourage primary care visits by clinics and health systems may be an effective way to improve compliance with preventative health interventions, and thus improve individual and population health.

## Data Availability

The data that support the findings of this study are available from Sanford Health but restrictions apply to the availability of these data, which were used under license for the current study, and so are not publicly available. Data are, however, available from the authors upon reasonable request and with permission of Sanford Health.

## Appendix

Propensity score methods used.

**Table 3:** Descriptives of patient characteristics and health care utilizations for 1,039,227 patients who potentially had vaccinations, 371,200 patients ages 50 and older who potentially had colonoscopies, 200,147 female patients ages 50 and older who potentially had mammograms, and 174,655 patients ages 65 and older who potentially had wellness visits

	Vaccinations *N* = 1,039,227		Colonoscopies *N* = 371,200		Mammograms *N* = 200,147		Wellness Visits *N* = 174,655	
	n	%	n	%	n	%	n	%
1 or More Vaccinations/Year	527,294	50.7						
No Vaccinations/Year	511,933	49.3						
1 Vaccinations/Year	187,126	18.0						
2 Vaccinations/Year	121,871	11.7						
3 Vaccinations/Year	114,959	11.1						
4+ Vaccinations/Year	103,338	9.9						
Colonoscopy			179,411	48.3				
Mammogram					135,933	67.9		
Wellness Visit							1659	1.0
1 or More Primary Care Visit/Year	471,077	45.3	177,068	47.7	98,296	49.1	84,540	48.4
No Primary Care Visits	568,150	54.7	194,132	52.3	101,851	50.9	90,115	51.6
1 PC Visit/Year	251,326	24.2	81,467	22.0	44,808	22.4	30,758	17.6
2 PC Visit/Year	108,517	10.4	49,607	13.4	27,281	13.6	24,955	14.3
3+ PC Visit/Year	111,234	10.7	45,994	12.4	26,207	13.1	28,827	16.5
Age Infant- 19	269,450	25.9						
Age 20–60	543,540	52.3						
Age 61–100	226,237	21.8						
Female	547,635	52.7	200,147	53.9			96,852	55.5
White	920,555	88.6	345,229	93.0	186,729	93.3	164,958	94.5
Urban	508,044	48.9	163,117	43.9	88,120	44.0	72,102	41.3
Large Rural	244,984	23.6	83,485	22.5	45,230	22.6	38,698	22.2
Small Rural	90,300	8.7	38,410	10.4	20,959	10.5	19,556	11.2
Isolated Rural	195,899	18.9	86,188	23.2	45,838	22.9	44,299	25.4
Current Smoker	141,180	13.6	50,811	13.7	24,615	12.3	13,257	7.6
Uses Alcohol	378,327	36.4	178,576	48.1	87,140	43.5	71,489	40.9
Had Hospitalization	130,238	12.5	58,847	15.9	30,116	15.1	36,298	20.8
Had Emergency Room Visit	42,370	4.1	20,217	5.5	13,245	6.6	8462	4.8
Used MyCare	305,681	29.4	112,360	30.3	68,896	34.4	44,813	25.7
	Mean	S.D.	Mean	S.D.	Mean	S.D.	Mean	S.D.
Vaccinations/Year	1.91	4.43						
Primary Care Visits/Year	0.91	1.46	0.99	1.49	1.04	1.53	1.15	1.66
Age	36.7	23.9	65.62	11.0	66.12	11.4	75.22	8.00
Propensity Score	0.47	0.15	0.47	0.16	0.45	0.16	0.47	0.16
Weight	2.00	0.71	2.00	0.77	2.00	0.82	2.00	0.77

The PSMatch procedure in SAS 14.2 was used for the estimation of propensity scores and weights. All variables were balanced between the two primary care groups and used in the propensity score. The inverse probability of treatment weighting is based on the propensity score to estimate average treatment effects. These weights were applied during the estimation of relative risks and the means differences tests to balance the effect of the covariates with primary care visits and control for confounding.

The numbers in this table are unweighted. Lightly shaded variables were the dependent variables for each of the four preventive health measures. The dark shaded variables were covariates used to create propensity scores for each preventive measure. All covariates were highly correlated (*p* < .01) with both primary care visits and the dependent variable of their measure. Age was used as a continuous variable in calculating the propensity score for analyses that weren’t sub-analyzed by age.

## Abbreviations

AWV: Annual wellness visit
USPSTF: US Preventative Services Task Force
